# Were the English Sweating Sickness and the Picardy Sweat Caused by Hantaviruses?

**DOI:** 10.3390/v6010151

**Published:** 2014-01-07

**Authors:** Paul Heyman, Leopold Simons, Christel Cochez

**Affiliations:** 1 Research Laboratory for Vector-Borne Diseases, Queen Astrid Military Hospital, Brussels B-1120, Belgium; E-Mails: pol.simons@telenet.be (L.S.); christel.cochez@mil.be (C.C.); 2 Reference Laboratory for Hantavirus infections, Queen Astrid Military Hospital, Brussels B-1120, Belgium

**Keywords:** English sweating sickness, Picardy sweat, hantavirus

## Abstract

The English sweating sickness caused five devastating epidemics between 1485 and 1551, England was hit hardest, but on one occasion also mainland Europe, with mortality rates between 30% and 50%. The Picardy sweat emerged about 150 years after the English sweat disappeared, in 1718, in France. It caused 196 localized outbreaks and apparently in its turn disappeared in 1861. Both diseases have been the subject of numerous attempts to define their origin, but so far all efforts were in vain. Although both diseases occurred in different time frames and were geographically not overlapping, a common denominator could be what we know today as hantavirus infections. This review aims to shed light on the characteristics of both diseases from contemporary as well as current knowledge and suggests hantavirus infection as the most likely cause for the English sweating sickness as well as for the Picardy sweat.

## 1. Introduction

Hantavirus infections, although only fairly recently intensively investigated, probably caused health problems for centuries. In Europe and Asia, Hemorrhagic fever with renal syndrome (HFRS) is an endemic zoonosis that infects around 100,000 individuals per year [1]. The causative agents are viruses of the genus *Hantavirus*, family *Bunyaviridae.* Rodents, insectivores and bats act as carriers. In rodents, hantaviruses are found in the *Cricetidae* family (subfamilies *Arvicolinae*, *Neotominae* and *Sigmodontinae*) and in the *Muridae* family (subfamily *Murinae*). Hantaviruses were also found in the *Soricomorpha* order, families *Soricidae* and *Talpidae*, in bats (order *Chiroptera*, families *Rhinolophidae*, *Nycteridae* and *Vespertilionidae*). Each hantavirus type is carried by a specific rodent/insectivore/bat host species and phylogenetic analysis revealed that the relationships between hantaviruses generally parallel the phylogeny of their rodent hosts. Recently, new hantaviruses have been found in insectivores and in bats [2], and the question with regard to the origin of hantaviruses was raised [3].

The earliest description of hantavirus infection dates back to China, around the year 900 AD. Hantavirus disease was suggested as a possible cause for the 1862–1863 “war nephritis” epidemic during the American Civil War, during which around 14,000 individuals developed a hantavirus disease-like condition [4,5]. An epidemic of trench nephritis during World War I may also have been caused by hantavirus infection [6,7,8]. In his attempts to identify the cause of the disease, Rutherford linked trench fever to voles, to the Sweating Sickness in England and to the Picardy Sweat in France: “*In the trenches field voles have been observed (as is only natural) lying dead in considerable numbers as the result of a gas attack … Field mice have been blamed as the source of a disease now extinct, the sweating sickness or sudor anglicus of the Middle Ages, of which a small epidemic, however, is said to have broken out in the south-west of France in the early years of the present century; it seems reasonable to suggest, in view of what has gone before, that they may perhaps be the source from which the infection of this new “trench fever” is derived as well.*” [6]. Raw and Tidy added to the argument by a description of, respectively, five cases and two cases that on hindsight closely match nephropathia epidemica (NE), the milder form of HFRS, infections caused by Puumala virus [8,9].

Since then hantavirus infections and hantaviruses have been detected and described on all continents except Australia [10] and are an increasing public health problem in many countries [1].

## 2. The English Sweating Sickness

The English Sweating Sickness, also referred to as Sudor Anglicus, English Sweat, the Sweat, the Swat, the New Acquaintance or “Stoupe! Knave and know thy master”, or “Stup-gallant” (both sarcastic names given by the poor, indicating that this new disease predominantly struck the rich), was and still is a historical and epidemiological mystery [11]. It struck midst the myriads of other diseases that almost constantly threatened the people’s health, but for which later on the causal pathogen was identified [12,13,14]. Particularly the medieval cities were so disease-ridden that they relied on a constant influx of people from rural areas to maintain the population at a constant level [15,16,17,18,19].

The English sweating sickness came in five major outbreaks, *i.e.*, in 1485, 1508, 1517, 1528 and 1551 [20,21,22,23,24,25,26]. A presumably more benign variant of the same disease, known as Picardy Sweat or “suette miliaire” was responsible for 196 outbreaks between 1718 and 1874 in Northern France. This form of the disease was never reported from the United Kingdom by most authors [27,28,29], although Mead mentioned a condition that he named “Dunkirk Fever” being brought to England towards the end the War of the Spanish Succession (1701–1714) by soldiers returning from mainland Europe in September 1712 [30,31], via the port of Dunkirk (*i.e.*, the Picardy region in N-France) [32,33,34]. The Dunkirk Fever proved much less deadly as none of the authors mention fatalities [35].

The Sweating sickness struck for the first time at the very beginning of the reign of Henry VII in 1485 [36,37,38], re-emerged in 1507, 1517, 1528 and made its last appearance in 1551. The five epidemics spanned the reigns of three British monarchs belonging to the House of Tudor, *i.e.*, Henry VII (1485–1509), Henry VIII (1509–547) and Edward VI (1547–1553) [39,40]. Webster however mentioned that—according to Erasmus—the English Sweat first appeared in 1483 and re-emerged in 1485 [41,42]. While, an account in the York Civic Records mentioned a pestilence in the area (N-E England) in June 1485 that bore great resemblance to the sweating sickness, *i.e.*, three months before the Battle of Bosworth Field [43].

The irregular intervals between the five epidemics (22, 10, 11 and 23 years respectively) suggest an ecological or meteorological trigger [44]. Its sudden onset and incredibly rapid course terrorized the citizens of 15th and 16th century England [20,21,22,23,24,25,26,45,46] and suggests a very common and abundant vector whose population peaked during those years, or human-to-human transmission.

Due to its sudden onset and its often fatal course, the English sweat became much feared and even Shakespeare expressed his concern for a re-emergence in his work “Measure for measure” (1604): “*Thus, what with the war, what with the sweat, what with the gallows, and what with poverty…*”. There also existed a prayer for protection against the Sweating Sickness, the document is currently preserved at Keio University, Japan [47], and in 1551 a complete liturgy “*A thankes geuing to God used in Christes churche*” was authorized, it was the first of a series of special worship liturgies in response to national disasters [48]. This divine protection was much needed as before the “Dissolution of Monasteries and Convents” (the closure of around 800 churches, friaries, priories and monasteries and often also the persecution of priests) by Henry VIII (1535–1539) the attack rate of the English Sweat amongst monks and the clergy was particularly high [49]. By papal decree (Pope Adrian I, 787) monasteries were entitled to a “tithe” (a tenth) of a person’s earnings or harvest. They thus often stored large quantities of food for long periods of time which might point to a rodent-borne source for the English sweat. Henry VIII decided to the “Dissolution of Monasteries and Convents” and initiated the *de facto* Protestant Reformation of England in consequence of his dispute with Pope Clement VII over the annulment of his marriage to Catherine of Aragon [50,51].

## 3. John Caius and Thomas Le Forestier

The most cited author with regard to the English sweat was Johannes or John Caius (John Kays, 1510–1573) [52]. But—as his birth year indicates—he was not an actual eye-witness of the Sweating Sickness epidemics except for the one in 1551. He left England in 1539 to study medicine and shared lodgings with Vesalius while studying in Padua, but supposedly assisted Thomas Gemimus in preparing his *Compendiosa* (1545), a pirated version of Vesalius’ *Epitome* (1543). Vesalius complained to Gemimus about “*a certain Englishman who aided in vitiating work*” [53,54]. He returned to England in 1545 and was the physician of King Edward VI, Queen Mary and Queen Elizabeth [52]. His famous and often cited work was thus mainly a summary of earlier published reports [20]. His work was also criticized by Creighton and Hecker [21], because his rhetoric only expressed grief for the loss of “noble lifes” and they doubted if he “really knew the facts about the disease in the country” [29].

Thomas Le Forestier on the other hand, was the only physician “on location” in England in 1485 and was active is fighting the disease [45,55]. Originally from Normandy (France), he left (or was obliged to leave) England two years later and published his works in Rouen, France, where he practiced medicine.

## 4. Setting the Stage for the English Sweating Sickness

In the 15th century, Europe underwent a subtle but dramatic change. Times were uncertain, life was hard and troubled by fear and superstition and it was preached that the “will of God” had brought plagues, earthquakes, meteorites, floods, droughts, famine, disease and war to the people [22,44,56]. The era between 1450 and 1550 was also one of the most important in European history. A far from complete list of “turning-point” events include the discovery of the New World (1492), the invention and spread of the printing press, the Copernican revolution and, last but not least, the Protestant Reformation (Lutheranism in Germany, Calvinism in Switzerland, and Anglicanism in England [57]). Most countries were also constantly involved in various armed conflicts which were responsible for deteriorated living conditions, famine, displacement and persecution of people. Only a few decades before the Black Death, sometimes named the biggest natural disaster that ever struck mankind, had plunged Europe into an economical recession, and caused social and agricultural destabilization [58,59,60]. The aftermath of the epidemic induced considerable ecological, economical and demographical changes (particularly by the redressing of abandoned land and forest to arable land) as with an again growing population, more food had to be produced [59,61]. Although the English sweating sickness was evidently a killer, the mortality data from contemporary sources have to be carefully interpreted as exaggeration was the rule in Medieval writings, the so-called “pestilence treatises” for instance became fashionable and the more horrible the content, the better they were sold [62]. The epidemics did not affect the population number the same way as did the plague. According to the demographic data the population of England decreased from 5.0 to 2.5 million in the 14th century, mainly due to the Black Death [63,64]. This was also true for mainland Europe where an estimated 17–28 million people succumbed to the disease between 1346 and 1353 [65]. But during the 15th and 16th century, and despite the English Sweat and various other epidemics, the English population grew from 2.5 to 4.4 million [64].

In this setting the War of the Roses was decided in England with a decisive battle—the battle of Bosworth Field [66], between the armies of Henry Tudor (Earl of Richmond, later king Henry VII, reigned from 1485 to 1509) and king Richard III (reigned from 1483 to 1485), who lost much support amongst the population due to his harsh ruling [22].

Henry Tudor, the Earl of Richmond undertook his second attempt to seize the crown of England in the summer of 1485. Henry Tudor received financial and logistical help from Charles VIII (king of France from 1483 to 1498), and a number of Welsh noble men promised support for overthrowing Richard III on English soil. His invasion army consisted for an important part of mercenaries. He disposed of the 2,000 mercenaries recruited by Louis XI, for a previous conflict and awaited the next in the region around Rouen, in the North-West of France (the very region where, in 1718, the Picardy sweat would emerge) [67]. The men constantly terrorized and looted the Rouen region—Shakespeare described them as “*A sort of vagabonds, rascals, and run-aways, A scum of Bretagnes, and base lackey peasants*” [68] (King Richard III, scene III, page 239)—and the French king was happy to lend them to Henry Tudor [69]. Mercenaries were the hard core of virtually every medieval army and Germany and Switzerland were reputed for providing soldiers of fortune. Although often identified as “French”, the mercenaries in Henry’s army were most likely not originally French but probably predominantly German and Swiss, with various smaller contingents of other nationalities probably present as well. The invasion force crossed the English Channel on the 7th of August 1485 and set foot on land in Milton haven, Pembrokeshire, Wales, besieged Dale Castle (Henry’s birth place) first and marched inland to join the promised local support [60,70].

## 5. The Five Epidemics

On the 22nd of August 1485, on Bosworth Field, Henry’s army defeated king Richard III and ended the Wars of the Roses [66]. The English Sweat already played a decisive role during this battle as one of the most influential lords of Wales—Lord Stanley, who contributed 30% of Richard III’s army—excused himself from the battle because his army allegedly suffered from “the sweat” although this did not prevent him from changing sides at the last moment and thus caused the balance to swing in favor of the invader, which resulted in the defeat and death of Richard III [66]. Almost immediately after the battle, a strange but deadly new disease broke out in the remainder of Henry’s army and followed it to London. Because of the regime change the city of London was probably full of people that planned to assist to the coronation of Henry and his allies came close to power. This temporary population increase may have contributed to the spread of the epidemic. A hallmark was its limitation to England, the local outbreaks were usually short and sharp, in London the epidemic raged until the 31st of October 1485 and took 15,000 lives [45].

Although the disease was allegedly “new” to Henry Tudor’s army, the fact that Lord Stanley already used “the sweat” as his excuse for not supporting Richard III, suggests that both the English king and Lord Stanley were aware of the existence of the disease in August 1485. Also the mention of the disease in 1483 by Erasmus and the report in the York Civic Records of a pestilence that resembled the English sweat three months before the invasion shed doubt on the importation theory from France to England by “French” mercenaries. This is further supported by several reports (see the chapter Origin and Epidemiology) that the English sweat was imported from Rhodes in or around the year 1480 [30,34,71,72,73]. Whether it was endemic on Rhodes in those days or imported by the Turkish invaders and thus originating from Asia Minor is not known.

In November 1485 the disease waned and did not re-emerge until 1508. The 1508 outbreak was less widespread, was confined to England and consequently took fewer lives. It appeared by the end of June and waned off by October [29].

The third epidemic occurred in 1517, again around the end of June and it was mainly confined to the London area. Not much is known about the event, but the Sweat was immediately followed by an outbreak of the plague in November 1517, which took apparently more lives than the Sweat [67,74,75].

In 1528–1529—(1528 on the English (Julian) calendar meant 1529 on the Roman (Gregorian) calendar), the dates in the text refer to the date in the location of the described event—the English sweat epidemic hit particularly hard and it was the most important outbreak as it spread to the European continent as far East as Russia [76,77]. Although it is assumed the disease appeared on the continent for the first time in 1529, Ozanam mentioned that already in 1517 the Sweat had victims in Antwerp (where it was named “zweetende” of “haestige ziekte” [78]) and France and there were also cases in Europe prior to 1528, *i.e.*, between 1525 and 1530 [79]. It is thus possible that the disease was endemic for an unknown period of time, but remained under the epidemic threshold, except during the five outbreaks. The Netherlands and The Flanders were affected in 1529 but France remained unaffected, except for the Calais region and the main port of entry of the region in those days, *i.e.*, Gravelines (in the proximity of Dunkirk, N-France). Italy and Spain also remained unaffected. The fourth outbreak started in London, where by the 30th of June it allegedly affected around 40,000 people but with only 2,000 deaths (5%) [29], it then spread across England but again stopped at the Scottish and Welsh Borders [80]. It then appeared in the Baltic and North Sea ports. In Antwerp [81,82], and Amsterdam [83,84] the disease emerged simultaneously on the 27th of September 1529; the outbreak lasted only four days, affected more than 2,000 individuals but the mortality seems to have been low [85,86,87].

Denmark [77], Sweden, Latvia, Lithuania, Poland, Switzerland [88,89] and Russia [90,91] were also severely affected but reports are rare or non-existent from Northern Europe.

In Germany, the disease claimed many victims [91,92,93,94]. Franz von Marval reports of the arrival of the Sweat in Hamburg by ship (he also mentioned the ship’s captain as being one Hermann Evers) on the 25th of July 1529, at least four people died of the Sweat in the city in the night following Evert’s arrival [87]. Again according to Franz von Marval, the ship transported many young people from England to Germany and at least 12 of them died underway in two days time [87]. Although probable, there is no mention of rodent presence, which might have led to the outbreak, on the ship. By the 30th of July the epidemic peaked in Hamburg and this course seems exemplary for most German cities (Lübeck, Mecklenburg, Bremen, Stuttgart, Heidelberg, *etc*.) where the disease broke out, *i.e.*, a sudden and steep rise of the number of cases and fatalities for four to six weeks after which the disease disappeared [95,96]. Von Marval also added a table to his thesis with the dates on which the disease struck German cities, the geographical distance between cities and the travel times (10 km on foot, 20–30 km by wagon or horse) in the 16th century might suggest human-to-human transmission as a plausible transfer method for the pathogen. The mortality rates were seemingly high, but did—as in England—not really affect the population as witnessed by, for instance, the Augsburg mortality bills, only the plague caused significantly higher mortalities [97].

The English sweat progressed from West to East, *i.e.*, in the opposite direction of influenza epidemics and it appeared in summer, while the plague was typically an autumn/early winter event. The forenamed countries were also hit by typhus and plague outbreaks on an almost yearly basis. This raises the question whether the Sweating Sickness was solely responsible for the high mortality that devastated Europe in 1528–1529. The report on the five percent mortality in London supports this assumption [29].

The last major outbreak (1551) brought terror again on England, but did—again—not cross the border with Scotland or Wales, nor the Channel to mainland Europe. The English sweat then disappeared, except for the mention of it in Colchester in 1578–1579 and in Rottingen in 1802.

There is only one mention of its appearance in Ireland (“pláigh allais” in Irish) in 1491 [98,99,100] and 1492 [101]. Although the reference is not extensive, the described symptoms closely match of what would become known as the English sweating sickness and there is the possibility that the 1491 Irish event heralded in fact the first emergence of the disease outside England. In “The Ancient and Present State of The County and City of Cork”, Charles Smith mentions the English sweat in Cork (S-Ireland) in 1528 [102]. Creighton further mentions an outbreak of the Sweat in Colchester in 1578–1579, *i.e.*, 27 years after what was considered the last of big outbreak of 1551 [29]. Another anomaly is the report of an outbreak in Rottingen, Germany, as late as 1802 which also strongly resembled the English sweating sickness [27].

## 6. Clinical Symptoms of the English Sweat

The onset of the disease was quick and without warning, according to contemporary descriptions usually during the night or early morning. The first symptoms were chills and tremors, quickly followed by high fever and a great weakness. The body was covered with perspiration and a rash was, contrary to the Picardy sweat, seldom reported. The course of the disease was exceptionally violent and sometimes fatal within hours. The mortality rate was highly variable but probably between 30% and 50% [29,103], Zinsser [104] cites a mortality rate as high as 80% to 90%, but Creighton reports a mortality of 5% [29]. The reason for the variability in mortality figures could have been the level of expertise of the physicians. Hamer described a number of them as: “*ignorant interlopers who with their pills and their hellish electuaries flit about from place to place, especially where rich merchants were to be found, from whom, should they be restored, they obtain the promise of mines of gold.*” [105]. The method for treating the patient does also not appear optimal in present time and probably contributed to the mortality, or as Creighton described it: “*the patients were placed instantly to bed, covered with clothes, windows being closed until the patient finally in his rehearsal of hell being bathed in an agonising sweat gave up the ghost*” [29,106,107]. Thomas More (1478–1535, councilor of Henry VII who fell out of grace and was beheaded after opposing to the King) described the disease as “more harmful than the sword”. Generally, the acute phase of the Sweat lasted for about twenty-four hours hence the name “*Ephemera Britannica*” [105], after which the patient recovered or died. Surviving for more than twenty-four hours generally indicated recovery and the perspiration was—as in hantavirus infections—replaced by polyuria [9,19,29,55,108,109]. Remarkably it also only seemed to affect Englishmen as there are no records of foreigners being affected on English soil [110,111]. Whether this is true or due to ignoring the faith of foreigners remains unknown, but the fact remains that several authors mention this [29,112]. Bordier even hypothesed rather convincingly that tall, fair-haired races were more vulnerable to the English sweat [110].

With regard to the incubation time of the English sweat, the most reliable source is Thomas Le Forestier [55] who stated that the sweat “*first unfurled his banners in England in the city of London, on the 19th of September*”, although Vitellius [112], and Sir Francis Bacon [113], gave different dates in September. Because there was mention of the disease in the troops of Henry during or after the arrival of the Army in Wales on the 7th of August and the Battle of Bosworth on the 22nd of the same month, the incubation time can be estimated to be from one day up to 29 days (from August 22nd to September 19th, if the Sweat only made victims at the time of the Battle of Bosworth). If the Sweat was already making victims when the invading army landed, the incubation time would be around 44 days (August 7th to September 19th) which is in line with the incubation time of hantavirus infections. The route the disease took coincided with that of the army, which could suggest human-to-human transmission. But, as armed forces in those days were accompanied by a caravan carrying supplies (including livestock), family, craftsmen, *etc*., the vector could have travelled along with the support troops.

## 7. Origin and Epidemiology

In contrast to most medieval epidemics—but in accordance to the hantavirus infection epidemiology—the English Sweating Sickness did not strike the young or old but the middle-aged, professionally active section of the population, especially wealthy, upper-class males [114]. This was so obvious, even at the time, that the poorer classes dubbed the disease the “*Stop Gallant*” [115]. Those of the poorer classes that were affected were described by Caius as idle persons; “*They which had this sweat sore with peril of death were either men of wealth, ease or welfare, or of the poorer sort, such as were idle persons, good ale drinkers and tavern haunters.*” [20].

The infection mode of the sweat remains unclear as neither the pathogen, nor the vector, were ever identified. According to some reports the disease was already present in England before 1485 [41,42,43] and it was suggested that in-between outbreaks there also occurred cases [81]. This suggests a rodent-borne disease, which typically shows seasonal as well as multi-annual fluctuations [116]. Although climatic conditions and events were carefully recorded throughout time [44], there exist—to our knowledge—no records of rodents or other wildlife species becoming pests. If the Sweat was rodent-borne, the black rat (*Rattus rattus*, present in Europe since prehistoric times) is—due to his synanthropic behavioral pattern—a prime candidate vector species and is known as a carrier of Seoul hantavirus.

Noteworthy is what Joseph Browne [30] wrote about the provenance of the Sweat: “you may find a description of it as coming from Hungary, by some troops sent thitter against the Turks by Henry VI, king of England”. Boott [71], repeats this and adds two more authors as reference, *i.e.*, Wedelius (Georg Wolfgang Wedel) and Mayerne (Théodore Turquet de Mayerne). Both coined the disease as “Febris Hungarica”, a denomination that was often used to name fevers of “tropical” origin, more precisely marsh fever, *i.e.*, yellow fever or malaria [117]. MacLean [73] goes into detail and wrote: “…and it was then thought to have been brought into France from the famous siege of Rhodes by the Turks, three or four years before (May to August 1480, the authors)” [34,73]. There may however be an overlap with the emergence of typhus fever (caused by Rickettsia prowazekii), the first appearance of which in Europe was during the siege of Granada in 1489–1492.The disease was often referred to as the “Hungarian disease” due to its upsurge during and after the Ottoman-Hungarian wars of the 15th and 16th century [118].

Contrary to the plague or smallpox that progressed steadily in the population and gradually made more and more victims, the English Sweating Sickness appeared and disappeared geographically at random. The duration of the respective outbreaks differed as did their violence; the third outbreak (in 1517) [76] was for instance far more violent than the second (in 1508), this outbreak it allegedly also reached across the English Channel and appeared at Calais, France where it only affected the Englishmen residing there. Evidently, there has been much speculation about the origin of the English sweat. Some contemporary scholars blamed the English climate, the moisture, the fogs, the way of living of the English people, and—as said before—the mercenaries of Henry VII. There were some solid arguments for the latter hypothesis as the German and Swiss “Landsknechte” were also held responsible for the spread of syphilis in Europe in 1495 [118]. The sweating sickness was an infectious disease, but different—as was observed by virtually all contemporary authors—from the plague, influenza, smallpox, typhus, scarlatina or malaria [119]. Human-to-human transmission was suggested, but is less likely due to the restriction of the disease in most epidemics to England despite intensive trade by ships, the usual vehicle of transmittable diseases like plague, between English and mainland Europe ports. Only in 1529 this seems to have been the case [87].

Medicine, then still considered to be and art, made in the 15th and 16th century considerable progress. Fracastoro, Vesalius and Paracelsus all suggested already that epidemics were caused by “objects living outside the body, that could be transmitted by direct or indirect contact”, and observations of clinical symptoms were rationalized [120,121,122,123]. Charles Creighton noted that the disease was harbored in the soils of the lower basin of the Seine, *i.e*., “endemic”, and that it was brought to England by Henry’s Flemish mercenaries (speculation, but “in case of doubt: blame a foreigner”), where disease ravaged in the immunological naïve population [29]. The geography might in this case point to the endemic hantaviruses in Northern France, Germany and Belgium but no hantavirus in Europe or Asia even comes close to being so violent and deadly. Moreover, there exist no reports of epidemics resembling hantavirus disease in the three forenamed countries at that time [21].

Nevertheless, as with all historical reports, some conservative interpretation with regard to the accuracy of the presented data and factual information is prudent; there exists for example a booklet by Churchyarde: “*The Miserie of Flaunders, Calamitie of Fraunce, Misfortune of Portugall, Unquietness of Irelande, Troubles of Scotlande; and the blessed State of Englande*.” that represents a rather amusing testimony of the particular state of mind in 16th century England with regard to “strangers” and their alleged shortcomings [124].

## 8. Possible Causes of the English Sweating Sickness.

After discarding meteorites, earthquakes, divine interventions, and other contemporary causes, a number of hypotheses remain to be discussed.

The hypothesis that the Sweating Sickness was a form of typhus was discounted because of the speed with which the symptoms appeared and the extremely short course of the disease [104,125].

Influenza was discounted because of the absence of any respiratory symptoms or secondary cases of pneumonia [126].

Recently, anthrax was suggested because of the rare symptoms observed during the 2001 inhalational anthrax cases in the USA, given the spore-forming capacity of anthrax (*Bacillus anthracis*) there might be a possibility of retrieving evidence of this hypothesis from Sweating Sickness victims in the 16th century [127].

The fact that the outbreaks were invariably preceded by a period of prolonged rainfall and, in some areas, extensive flooding [44] then led to the presumption that an arbovirus (tick- or mosquito-borne) could be the cause, which could explain why the higher, colder parts of the British Isles (Scotland, Wales) remained unaffected. In the tick-borne hypothesis, the more virulent Russian spring-summer encephalitis and Omsk Hemorrhagic fever were suggested [25]. Carlson and Hammond [128] suggested Crimean-Congo Hemorrhagic Fever (CCHF) as a candidate, the geographical restriction of the outbreaks however is with CCHF as causal agent difficult to explain.

Rickettsial pox was considered a candidate in line with symptoms observed during the Queens outbreak (New York City) in 1946 [129,130].

The food-borne botulism hypothesis—eating fish and Swabian sausages was blamed in Germany—was suggested, but seems, again due to the geographical restrictions of the disease, unlikely [115]. Further proof was unfortunately not available for none of the hypotheses and all authors concluded the true cause lies hidden in the mist of time.

A plausible explanation for the restriction of the English sweat to England was suggested by Millingen and Willan [131,132], *i.e.*, according to both authors the English sweat was caused by a specific, but common food poisoning in the Middle Ages, a fungus (*Claviceps purpurae*) that grows on grasses and cereals and its occurrence has been linked to cold winters followed by wet summers. It contains certain alkaloids (e.g., ergocryptin) that produce a condition that is called ergotism in humans and animals. Poisoning by ergot was rather common in the Medieval era in Europe [133]. Millingen blamed cereals (wheat, rye), the main ingredient of the bread of the English in those days [131] for the limitation of the English sweat to England and argued that the Scottish and Welsh in contrast consumed mainly barley and oats [134,135]. A food-borne cause was later on judged unlikely, mainly because rye cultivation was far less popular in England than on mainland Europe [133]. An anonymous writer who called himself The Inquirer [136], opposed to the ergotism hypothesis and pointed out that ergotism occurred in years where the rye and wheat harvest failed and cereals became rare and expensive. Famine, the direct consequence of scarcity was, according to him, not recorded in the years that the Sweat raged in England and also one of the prominent symptoms of ergotism, *i.e*., gangrenous manifestations in the limbs were not recorded in the course and outcome of the English Sweat [136,137]. Creighton however, wrote exactly the opposite and mentions years with wet winters, heavy rainfall and destroyed harvests in the years preceding the Sweat epidemics [29]. An insect or rodent source that has a link with the cereal harvest should however be considered. The timing of the successive epidemics, *i.e.*, summer to beginning of autumn, and the circumstances, *i.e.*, years with harsh winters and with heavy rain fall in spring, supports this assumption and this could in turn point to a mosquito-, tick-, but also a rodent-borne disease. If a hantavirus is considered as cause, insectivore population peaks due to the availability of insects in summer or increased bat activity, again due to the high seasonal prevalence of flying insects, should also be taken into account.

Finally, in 1997, it was suggested that the English Sweat was caused by a medieval ancestor of the hantavirus species which has recently appeared in North America and provoking Hantavirus pulmonary syndrome (HPS) [138,139]. The clustering of the cases, the pulmonary components as described by Le Forestier [45,55] and the outbreaks after abundant rainfall in fact resemble the epidemics caused by population fluctuations of infected peridomestic small mammal populations [140]. However, if the English sweating sickness had been caused by a hantavirus, at least isolated cases would have been reported over time in England and this has not been the case. When comparing English sweating sickness, the Picardy sweat, HPS and HFRS, there is reason to suggest that Sweating sickness could also have been caused by an Old world hantavirus (with an *Arvicolinae* or *Muridae* rodent as carrier) (Table 1), which would be in line with the more benign nature of the Picardy Sweat in Northern France in the 18th and 19th century. Why the virus was so much more virulent in the 15th–16th century cannot be explained by genetic variation in present-day hantaviruses [141]. The Dobrava hantavirus complex however, in which different genotypes–although genetically highly similar—give very different clinical pictures [142], might point to a similar situation for the virus (on the condition it was a virus, and a hantavirus) that caused the English sweat and the Picardy sweat. Also, due to the recent discovery of the presence of hantaviruses in insectivores and bats [2], the possibility of an insectivore or bat-borne hantavirus should be taken into account [3].

**Table 1:** Comparison of the English sweating sickness, the Picardy sweat, HPS and HFRS.

	English Sweating Sickness *	Picardy Sweat **	HPS ***	HFRS ****
Vector	?	?	Rodent	Rodent
Pathogen	? (virus?)	? (virus?)	Hantavirus	Hantavirus
Infection mode	? (zoonotic?; human-to-human?)	? (zoonotic?; human-to-human?)	Aerosol	Aerosol
Incubation time	1–44 days	6 days ^§^	1–40 days	1–40 days
Disease stages	Headache, myalgia, sweating Abdominal pain, vomiting Delirium Cardiac palpitation, Breathlessness Convalescence/death	Febrile phase, sweating Hemorrhages: rare, but violent Rash (redness of the skin): common Convalescence/death	Febrile phase Pulmonary edema, shock Diuresis Convalescence/death	Febrile phase Shock Oliguric phase Diuretic phase Convalescence/death
Duration	24 h (if fatal)	10–14 days	10–20 days; 2–5 days, if fatal	10–20 days
Mortality	30%–50%	0%–20%	40%	<1%
Seasonality	Summer	Summer	Summer	Spring, summer

HPS: hantavirus pulmonary syndrome; HFRS: hemorrhagic fever with renal syndrome; * According to [20,45,55]; ** According to [106,143,144]; *** According to [139,141]; **** According to [1,141]; § According to [145].

## 9. The Picardy Sweat

The only disease which bears resemblance to the English Sweating Sickness is the *Picardy sweat*, *also called miliary fever*, *in French* “*suette des Picards*”, *or* “Frieselfieber”, in German*.* Although miliary fever was mostly confined to France (but certainly not only to the Picardy region), it also caused outbreaks in Germany, Belgium, Austria, Switzerland and Italy. Noteworthy is that Wolfgang Amadeus Mozart’s cause of death (he died in Vienna, on the 5th of December of 1791) was originally described as “hitziges Frieselfieber” or “severe military fever” [146,147]. The disease emerged for the first time in north-west France in 1718 and caused in total 196 outbreaks, which were mostly localized and much more benign, up until 1861 [26]. In spite of these published dates, several outbreaks have been reported in other parts of France and neighboring countries and also after 1861 several outbreaks were mentioned in various reports. It was also characterized by intense sweating but was less fatal [148], it occurred in limited epidemics of short duration in summertime [108,143,144,149,150,151]. The Picardy sweat was a predominantly rural disease that struck small villages [152]. Chantemesse, who presented a remarkably detailed epidemiological account of the outbreak, was very specific about the restriction of the outbreak to the rural area and even prolonged visits of ill individuals to the city of Rouillac (situated in the department Charente, between Cognac and Angoulême, *i.e.*, approximately 700 km south of the Picardy region) did not induce illness in the city, which does not support the thesis of human-to-human transmission [152]. Two different forms were described, *i.e.*, a benign form that strongly resembles nephropathia epidemica and a severe form that resembled the English sweat [145]. The same author also named the causal pathogen “a virus that came from the fields” and observed that the individuals that slept closest to the ground were most likely to be infected. Based on the these observations Chantemesse concluded that transmission occurred through flea bite and was related to the plague, although the symptomatology did not match and the causal pathogen (*Yersinia pestis*) is a bacteria. He then linked the outbreak to observations of rodent (probably voles) invasion of homes after flooding [152]. Mortality during the Rouillac outbreak ranged between 0% and 20%. In that respect, one could speculate that the Picardy Sweat had time on its side as it appeared much later than the English Sweating Sickness. The concept of disease had indeed evolved by that time from the medieval humoral/astrological to the observational approach which was without doubt to the benefit of the patient and the outcome of the disease [11,144,153]. A popular treatment against the Picardy sweat in the 18th century was the administration of quinine sulphate in doses as high as three grams a day, which apparently gave good results [108,144,154], although various experiments with other treatments existed [154]. Also remarkable was the use of a disinfection apparatus in disease ridden villages: “*a sanitary train that was very successfully used during the prevalence of an epidemic of sudor Anglicus in Poitou this year*” (the year is not specified, the reference to the English sweat—“*sudor anglicus*”—is not further elaborated, but the time of its usage puts it at the end of the Picardy sweat era, *i.e.*, early 19th century [155]*.*

Finally, the most recent mention of “sudor anglicus” dates from occupied France during World War II, in which a health risk warning against “sudor anglicus” is issued for the Vienne valley (N-E France), today the Southernmost region in France where hantavirus infections occur. The report was summarized in a Dutch journal and mentions that the disease affects primarily people between the ages of 20 to 40 years [156].

If a hantavirus was involved in the Picardy sweat, the causal virus might have been a pre-Puumala or Puumala variant instead of the unknown, apparently disappeared virus that struck England. But contrary to a hantavirus as the cause of the English Sweating Sickness, the Picardy Sweat could have been easily caused by a hantavirus as Puumala infection still occurs in and around the same region today. Chantemesse linked the occurrence of the Picardy sweat to population peaks of what he called “le rat des champs”, in French an ambiguous name that can refer to several rat or vole species, but most likely to *Microtus agrestis* [152]. Keeping in mind the high physical resemblance between bank voles and field voles, this might again lead to Puumala virus.

## 10. Conclusions

It is evident that all argumentation—without evidence for the causal agent of the English sweating sickness or the Picardy sweat—is mere speculation and circumstantial evidence; not enough to “convict” a hantavirus. The mystery around the origin of the English sweating sickness is perhaps best worded by a quote of Sherlock Holmes: “*Once you eliminate the impossible, whatever remains, no matter how improbable, must be the truth*”.

While all plausible causes, *i.e.*, influenza, thypus, plague, arboviruse(s), botulism, ergotism, *etc.*, were one by one discarded, hantavirus infection remains a strong possibility based on the resemblance of the Sweating sickness with HPS (or HFRS with pulmonary involvement). The very early conclusion that the Sweat was different from all “plagues” known at that time, the seasonality and the climatic features that apparently preceded the five epidemics, all point at least to another-than-the-usual-candidates pathogen, viral and vector-borne. A major drawback is the fact that in modern times only very few hantavirus infections were described in the United Kingdom although there has recently been an “upsurge” in hantaviruses in the United Kingdom [157,158]. It is also not unlikely that a virus harbored by a nice carrier species, be it rodent, insectivore or bat, has escaped detection so far [159]. It was and still is also widely accepted that the Picardy sweat resembled the English sweat and was probably “of the same nature”. Linking the Picardy sweat to a hantavirus is however much more easy as hantavirus infections are well known in Northern France in the 20th and 21st century. There was a gap of 150 years between the English sweat and the Picardy sweat, while between the Picardy sweat and hantavirus epidemics today there is again a gap of more than 100 years, only in periods of great turmoil (WWI, WWII) epidemics that resemble hantavirus infection are to have taken place. As much as hantaviruses and hantavirus infections of today are still under debate, the nature and origin of the English Sweating disease and the Picardy Sweat are both still medical mysteries and will most probably remain so [160]. In an era where many new viruses emerged, e.g., SARS, HIV, MER-coV, *etc.*, the importance of the lessons-learned from the English sweating sickness and the Picardy sweat should however not be underestimated.
